# Identification of *StbZIP* in Potato (*Solanum tuberosum* L.) and *StbZIP104* Enhances Cold Resistance

**DOI:** 10.3390/plants15101513

**Published:** 2026-05-15

**Authors:** Yihan Zhao, Chunna Lv, Yifan Zhou, Rong Li, Yuting Bao, Minghao Xu, Fang Wang

**Affiliations:** 1 Academy of Agriculture and Forestry Sciences, Qinghai University, Xining 810016, China; s8275069888@126.com (Y.Z.); lcn2922022@163.com (C.L.); zhouyifan200109@163.com (Y.Z.); rongli_ya@163.com (R.L.); baoyuting0318@163.com (Y.B.); m18697239510@163.com (M.X.); 2 Laboratory for Research and Utilization of Qinghai Tibet Plateau Germplasm Resources, Qinghai University, Xining 810016, China; 3 Qinghai Provincial Key Laboratory of Potato Breeding, Qinghai University, Xining 810016, China

**Keywords:** bZIP family, cold tolerance identification, low-temperature stress, tetraploid potato

## Abstract

Low-temperature stress significantly limits plant growth, development, and productivity, posing a major environmental constraint. The potato (*Solanum tuberosum* L.) is particularly vulnerable to low temperatures, underscoring the crucial need to enhance cold tolerance in potato breeding efforts for sustainable production. Basic leucine zipper (bZIP) transcription factors serve as central regulators of plant developmental processes and stress responses; however, their functional role in cold tolerance in tetraploid potato remains poorly understood. Here, we report a systematic characterization of the *bZIP* gene family in tetraploid potato and provide preliminary evidence that *StbZIP104* enhances plant cold tolerance. A total of 191 *StbZIP* genes were identified and classified into 11 subfamilies, exhibiting uneven chromosomal distribution and expansion primarily driven by whole-genome and segmental duplication. Promoter cis-element analysis, together with GO and KEGG enrichment analyses, indicated that *StbZIP* genes are broadly associated with hormone signaling, stress responses, signal transduction, and environmental adaptation. Expression profiling under low-temperature treatment revealed eight cold-inducible *StbZIP* genes (log_2_FC ≥ 1 and FDR < 0.05), among which *StbZIP104* was strongly induced (log_2_FC ≥ 2) and showed 5.36-fold higher expression in highly cold-resistant cultivars than in cold-sensitive cultivars. Subcellular localization confirmed that *StbZIP104* is a nuclear-localized protein. Functional validation confirmed that overexpressing *StbZIP104* notably improved cold tolerance in transgenic *Samsun NN tobacco (Nicotiana tabacum cv. Samsun NN)*. This was supported by heightened superoxide dismutase and peroxidase activities, increased levels of soluble protein and soluble sugars, and decreased malondialdehyde content compared to the wild type under cold stress. This study establishes a basis for the functional characterization of the *bZIP* gene family in tetraploid potato and serves as a theoretical reference for understanding the mechanisms that govern cold tolerance in this species.

## 1. Introduction

Potato (*Solanum tuberosum* L.), a crop with a high yield potential, provides food security for many countries in the world. Potatoes are particularly vulnerable to low-temperature stress [1], which causes stem lodging and water-soaked, dark-green leaves, leading to a loss of photosynthetic capacity, growth arrest, and ultimately yield reductions [2,3]. China has four major potato cultivation regions with distinct cropping systems, all of which are affected by low-temperature stress to varying degrees [4]. The northern single-cropping region and the central double-cropping region are vulnerable to late spring cold events and early autumn frost, which may lead to yield losses. The southwestern mixed-cropping region is mainly located in low-mountain and river valley areas at elevations of approximately 1000–2000 m; cold waves or strong southward-moving cold air masses are often accompanied by frost and strong winds, thereby affecting local potato production. In the southern winter-cropping region, potato cultivation mainly relies on winter fallow fields, and low temperatures during winter and early spring can markedly influence potato growth and yield.

The bZIP transcription factors, a highly diverse family widely present in plant genomes, consist of a conserved domain containing a basic region and a leucine zipper region [5]. The leucine zipper domain facilitates dimerization, while the basic region enables binding to specific DNA sequences [6]. Extensive studies have shown that bZIP transcription factors regulate plant growth and development while mediating responses to both biotic and abiotic stresses [7]. Notably, bZIP transcription factors are essential regulators of low-temperature responses. For example, *AtbZIP60* has been reported to enhance cold tolerance in Arabidopsis thaliana by upregulating the expression of calcium-dependent protein kinase (CDPK) genes [8]. Additionally, *SlAREB1* improves cold tolerance in tomato by promoting anthocyanin accumulation [9]. Transcriptome analyses have indicated that *ZmbZIP68* negatively regulates cold tolerance in maize by repressing *ZmDREB1* expression [10]. However, investigations of the bZIP family in the tetraploid potato remain limited, particularly with respect to expression dynamics under low-temperature stress.

Although the significance of bZIP family in plant stress responses is well established, the composition and cold-responsive functions of the bZIP family in tetraploid potato are not fully elucidated. Here, we systematically identified and characterized the bZIP family in the tetraploid potato cultivar ‘Qingshu 9’. Our analysis encompassed the physicochemical properties, phylogenetic relationships, gene structures, conserved motifs, promoter cis-acting elements, and chromosomal distribution of *StbZIP* members. We further examined their expression profiles using published RNA-seq datasets and compared the expression of selected *StbZIP* genes under low-temperature treatment among potato cultivars with contrasting cold tolerance. Based on these analyses, *StbZIP104* was identified as a cold-responsive candidate gene. Subsequently, overexpression lines were generated to preliminarily validate that *StbZIP104* enhances plant cold tolerance. This study lays the groundwork for additional functional characterization of the *bZIP* gene family in tetraploid potato and serves as a theoretical reference for understanding the mechanisms involved in cold tolerance in potato.

## 2. Results

### 2.1. Genome-Wide Identification, Phylogenetic Classification, and Chromosomal Distribution of the StbZIP Family

**Figure 1:**
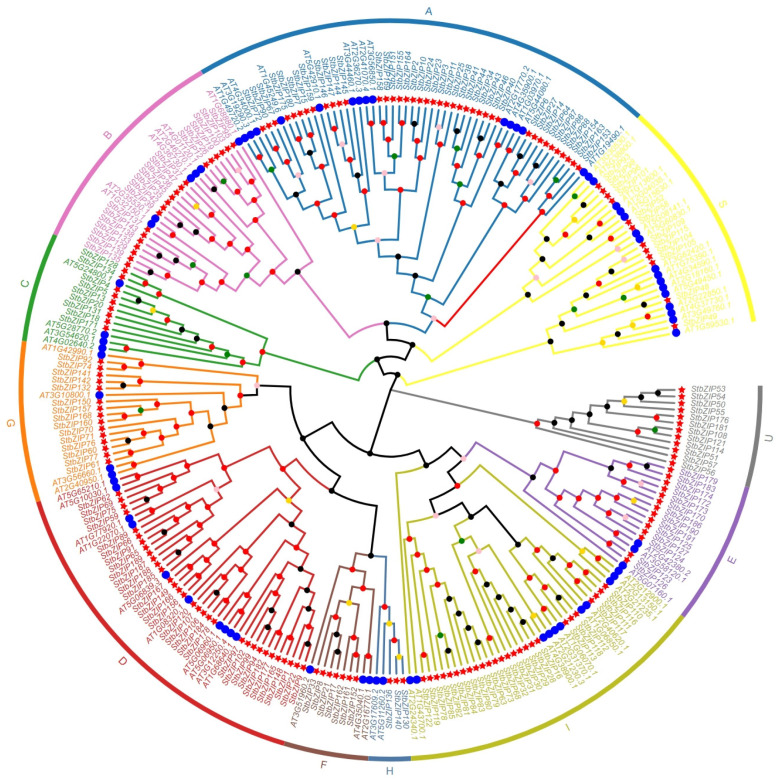
Phylogenetic relationships of bZIP proteins from tetraploid potato and Arabidopsis. The blue circles in the inner ring represent bZIP proteins from Arabidopsis thaliana, whereas the red pentagrams indicate bZIP proteins identified in potato. Distinct hues in the outer circle indicate various subfamilies. The sequence of subfamilies, beginning with the yellow sector, is as follows: S, A, B, C, G, D, F, H, I, E, and U. Colored dots on the branches indicate bootstrap support values: dark gray, 0–0.20; pink, 0.21–0.40; gold, 0.41–0.60; green, 0.61–0.80; and red, 0.81–1.00.

A total of 191 *bZIP* genes were identified in the tetraploid potato. Multiple sequence alignment with Arabidopsis thaliana bZIP proteins followed by phylogenetic reconstruction yielded a tree in which the StbZIP family was classified into 11 subfamilies (A–I, S, and U) (Figure 1). Subfamily sizes ranged from 3 to 41 genes, with subfamily A being the largest and subfamily H the smallest. The number of members in subfamily H was comparable between potato and Arabidopsis, suggesting a relatively high degree of evolutionary conservation. Notably, the U subfamily was observed exclusively in potato.

**Figure 2:**
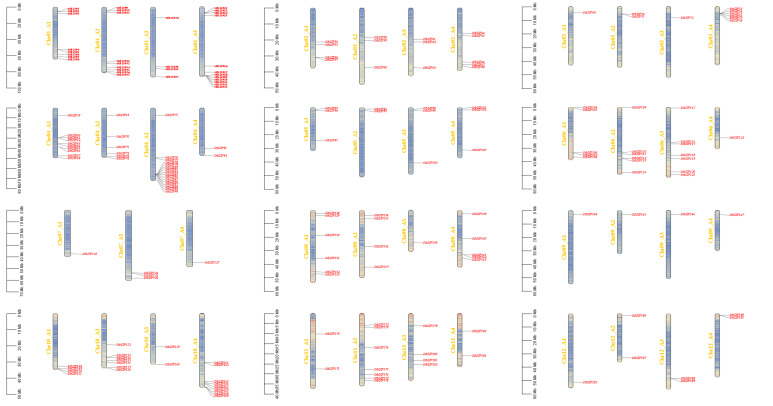
The distribution of bZIP genes in potato chromosomes (red color).

Chromosomal mapping (Figure 2) revealed an uneven distribution of *StbZIP* genes across the 47 chromosomes. Notably, no *StbZIP* genes were found on Chr07A2, while Chr04A3 contained the highest number of genes (16).

The 191 bZIP proteins exhibited considerable variation in relative molecular mass (9493.52–95,535.82 Da), isoelectric point (4.24–10.22), and length (83–862 amino acids). DeepLoc predicted that the majority of these proteins localize to the nucleus (177), while additional proteins were assigned to the endoplasmic reticulum (7), to both the nucleus and endoplasmic reticulum (5), or to the cytoplasm and nucleus (2) (Table S1).

### 2.2. Gene Structure, Conserved Domains, and Conserved Motifs of the StbZIP Family

**Figure 3:**
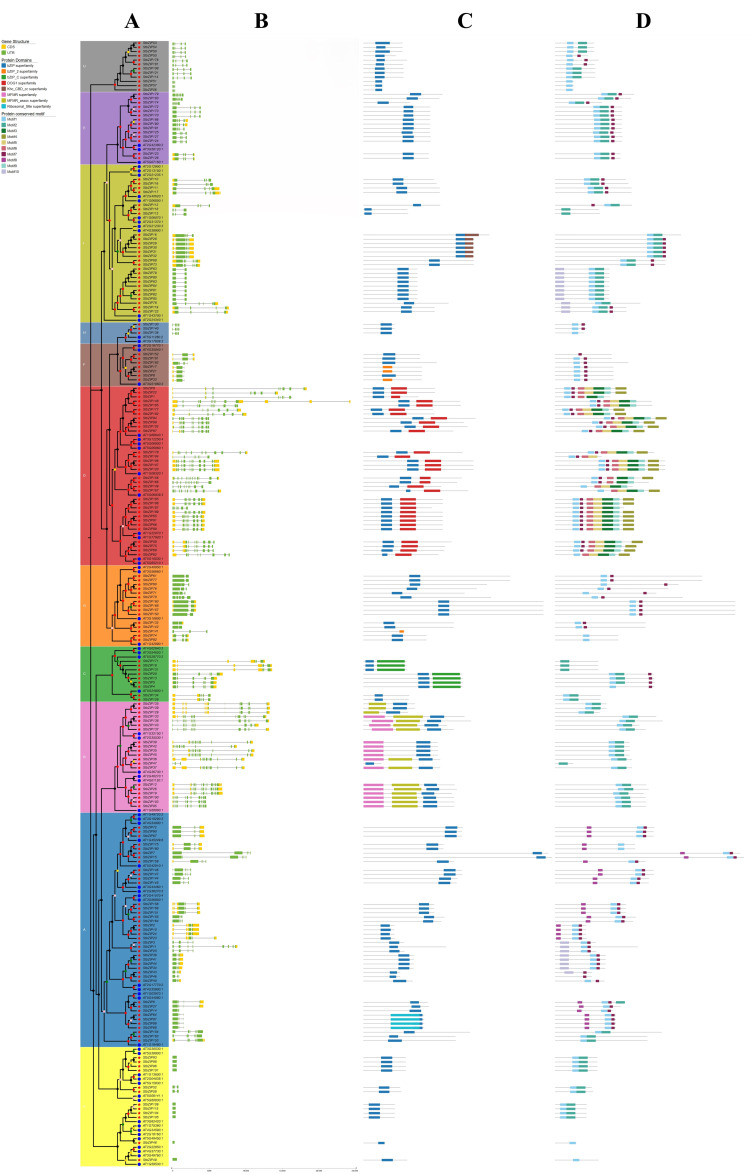
**Gene structure (B), domain (C), and conserved motifs (D) of the StbZIP family.** (**A**) Phylogenetic relationships of StbZIP proteins. The blue circles in the inner ring represent bZIP proteins from Arabidopsis thaliana, whereas the red pentagrams indicate bZIP proteins identified in potato. Distinct hues in the outer circle indicate various subfamilies. The sequence of subfamilies, beginning with the yellow sector, is as follows: S, A, B, C, G, D, F, H, I, E, and U. Colored dots on the branches indicate bootstrap support values: dark gray, 0–0.20; pink, 0.21–0.40; gold, 0.41–0.60; green, 0.61–0.80; and red, 0.81–1.00. (**B**) The exon–intron organization of the *StbZIP* genes is illustrated, with yellow boxes indicating exons, black horizontal lines representing introns, and green boxes denoting untranslated regions; (**C**) the conserved domain analysis of the StbZIP family; (**D**) the conserved motif analysis of the StbZIP family.

Gene structure analysis (Figure 3B) revealed that *StbZIP* genes vary in length from 294 to 24,358 bp and contain between 2 and 14 exons, with members of the same subfamily displaying similar exon–intron architectures. Conserved-domain profiling (Figure 3C) demonstrated that all proteins possess a bZIP superfamily domain. The DOG1 superfamily domain was detected exclusively in subfamily D, while bZIP_C superfamily domains were found only in subfamily C. Additionally, MFMR and MFMR-assoc. superfamily domains were identified solely in subfamily G. A total of ten motifs (Motif 1–Motif 10) were identified, and only six proteins within the StbZIP family were devoid of Motif 1. Motif 3, Motif 5, and Motif 6 were confined to subfamily D (Figure 3D).

### 2.3. Distribution and Functional Classification of Cis-Acting Elements in StbZIP Promoters

**Figure 4:**
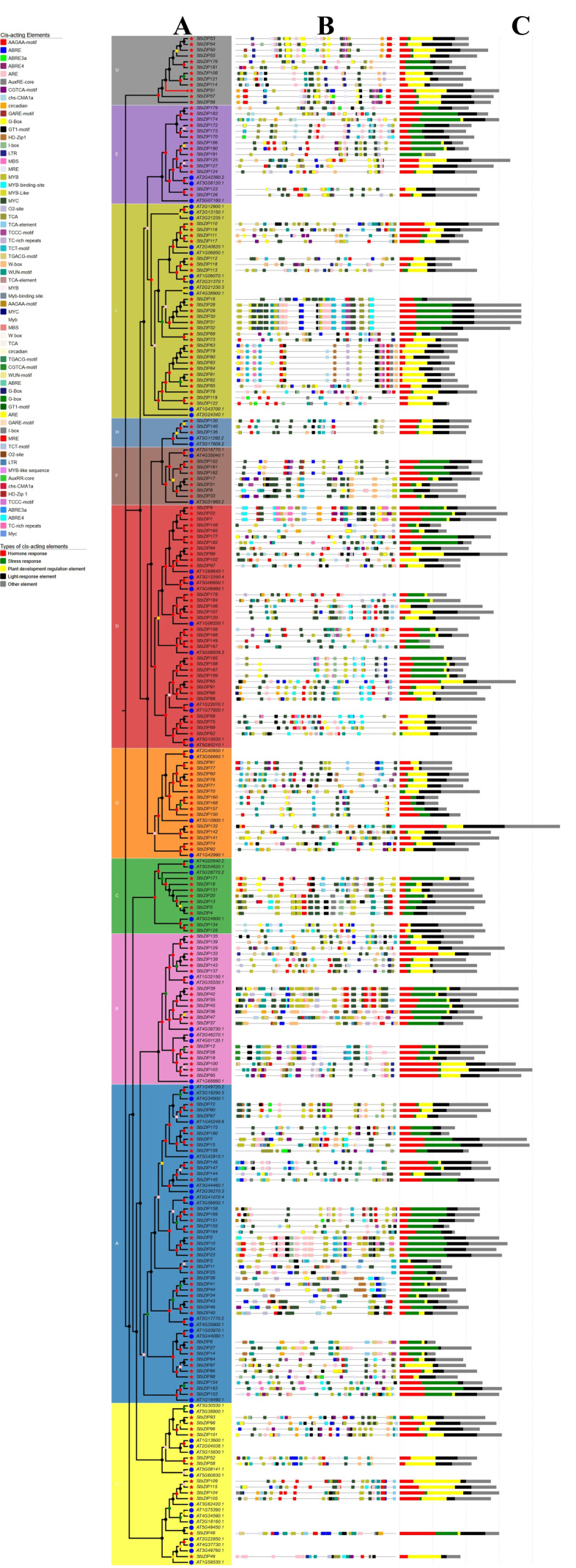
**Cis-acting element analysis in the promoter regions of *StbZIP* genes.** (**A**) Phylogenetic relationships of StbZIP proteins. The blue circles in the inner ring represent bZIP proteins from Arabidopsis thaliana, whereas the red pentagrams indicate bZIP proteins identified in potato. Distinct hues in the outer circle indicate various subfamilies. The sequence of subfamilies, beginning with the yellow sector, is as follows: S, A, B, C, G, D, F, H, I, E, and U. Colored dots on the branches indicate bootstrap support values: dark gray, 0–0.20; pink, 0.21–0.40; gold, 0.41–0.60; green, 0.61–0.80; and red, 0.81–1.00. (**B**) Cis-acting element analysis in the promoter regions of *StbZIP* genes. (**C**) The classification of promoter cis-acting elements of the *StbZIP* genes.

Thirty distinct cis-acting element types were identified and classified into five functional classes based on their responses: hormone responsive, stress responsive, light responsive, development related, and “other”. (Figure 4) Among the detected elements, excluding the “other” category, abscisic acid-responsive elements (ABRE, ABRE3a, and ABRE4) were the most prevalent, totaling 433. Notably, 73.82% of *StbZIP* gene promoters contained hormone-, stress-, and light-responsive elements, suggesting a broad regulatory capacity in signal transduction and environmental responses.

### 2.4. Duplication Patterns, Selection Pressure, and Syntenic Relationships of the StbZIP Family

**Figure 5:**
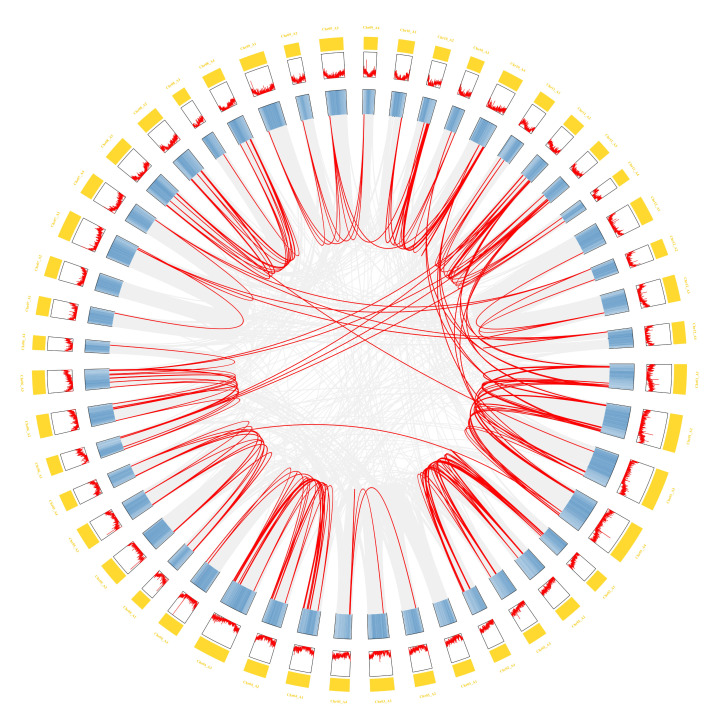
**Chromosomal distribution and synteny relationships of StbZIP genes in tetraploid potato.** From the outside to the inside, the outermost segments represent chromosomes, the histogram tracks indicate the distribution of StbZIP genes, and the inner tracks indicate chromosome regions/gene-density distribution. A total of 191 *StbZIP* genes were mapped onto 47 chromosomes of the potato cultivar ‘Qingshu No. 9’. The gray lines in the background indicate collinear blocks within the ‘Qingshu No. 9’ genome, whereas the red lines represent collinear *bZIP* gene pairs.

To clarify the gene duplication events responsible for the StbZIP family, we conducted an intraspecific synteny analysis in tetraploid potato. Across the genome, *StbZIP* genes displayed significant syntenic connections (Figure 5). In total, 226 syntenic gene pairs were identified, including 214 pairs derived from whole-genome/segmental duplication (WGD/segmental) and 12 pairs from tandem duplication (Table S2). These findings suggest that WGD/segmental duplication has primarily propelled the enlargement of the StbZIP family.

The selective pressure acting on the StbZIP family was evaluated by estimating the nonsynonymous substitution rate (Ka), synonymous substitution rate (Ks), and Ka/Ks ratio for 226 duplicated gene pairs (Table S2). Most pairs (205/226) exhibited Ka/Ks < 1, indicating that the tetraploid potato bZIP family has evolved predominantly under purifying selection. In contrast, eight pairs showed Ka/Ks > 1, suggesting episodes of positive selection. For 13 duplicated gene pairs, Ka/Ks values were not obtained, possibly because synonymous and nonsynonymous substitution values were unavailable for these gene pairs in the genome [11]. The mean Ka/Ks value of WGD/segmental duplicated pairs was 0.3366, whereas tandem duplicated pairs had a slightly higher mean Ka/Ks of 0.3717.

**Figure 6:**
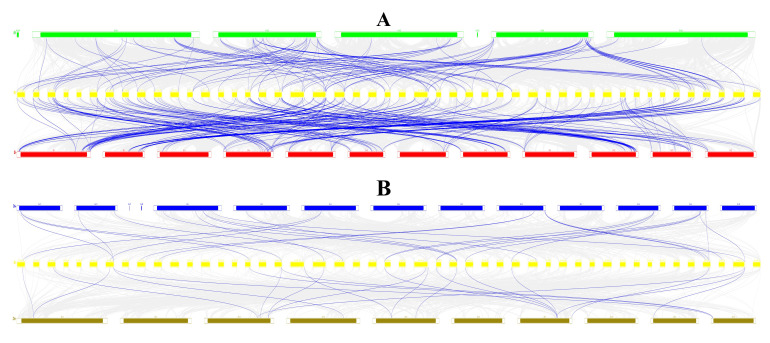
**Collinearity analysis of *bZIP* genes among potato and various species.** The gray lines in the background represent the collinear blocks between the potato and the other four plants, the red lines indicate the collinearity of *bZIP* pairs. (**A**) Arabidopsis (At-, green) is shown at the top, the tetraploid potato (St-, yellow) in the middle, and tomato (Sl-, red) at the bottom. (**B**) Rice (Oz-, bule) is shown at the top, the tetraploid potato (St-, yellow) in the middle, and maize (Zm-, brown) at the bottom.

Species-level synteny analyses were performed between the tetraploid potato and Arabidopsis thaliana, tomato (*Solanum lycopersicum*), rice (*Oryza sativa*), and maize (*Zea mays*) to investigate the evolutionary context of the StbZIP family (Figure 6). A total of 103, 219, 26, and 21 collinear *bZIP* gene pairs were identified in the potato–A. thaliana, potato–tomato, potato–rice, and potato–maize comparisons, respectively. The substantially higher number of syntenic pairs between potato and tomato highlights the strong collinearity and evolutionary conservation of *bZIP* genes within *Solanaceae*. By contrast, fewer syntenic pairs were detected between potato and rice or maize, suggesting that only a limited number of conserved *bZIP* syntenic relationships were retained after the divergence of dicots and monocots. These results provide evidence for both the conservation and lineage-specific divergence of the *bZIP* family during plant evolution.

### 2.5. GO and KEGG Enrichment Analysis of the StbZIP Family

**Figure 7:**
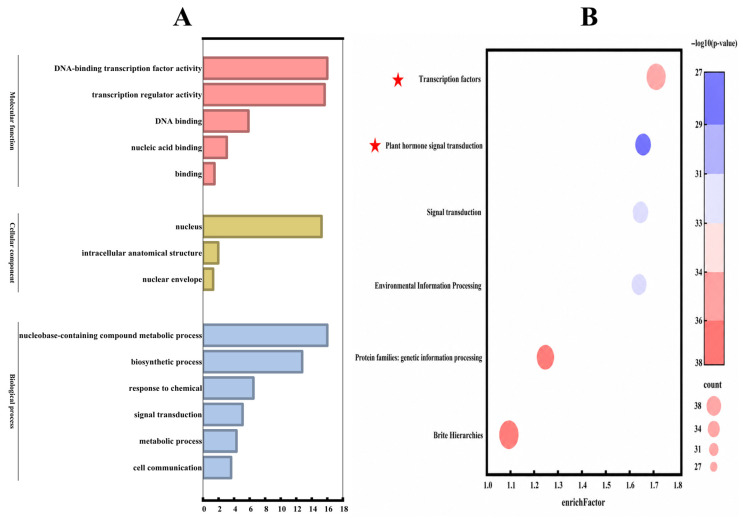
**GO and KEGG enrichment analysis of the StbZIP family.** (**A**) The GO enrichment analysis of the StbZIP family; (**B**) the KEGG enrichment analysis of the StbZIP family. The rich factor on the *x*-axis represents the ratio of the number of differentially expressed genes enriched in a given pathway to the total number of genes annotated in that pathway. Dot color indicates enrichment significance, represented as −log10 (*p*-value), whereas dot size corresponds to the number of genes enriched in each pathway. The pathways marked with red pentagrams are considered key pathways.

GO enrichment analysis (Figure 7A) was summarized across the three GO domains: molecular function (MF), cellular component (CC), and biological process (BP). In MF, DNA-binding transcription factor activity and transcription regulator activity were significantly enriched, indicating a strong concentration of transcriptional regulatory functions within the StbZIP family. In CC, nucleus was the predominant enriched term, consistent with the nuclear localization of these regulators. In BP, nucleobase-containing compound metabolic process and biosynthetic process were prominently enriched, suggesting involvement in nucleic acid metabolism and biosynthesis. Moreover, enrichment for “response to chemical” and “signal transduction” was detected, supporting putative roles of *StbZIP* genes in environmental stimulus responses and plant signal transduction pathways. KEGG analysis (Figure 7B) identified the “Transcription factors” pathway as the most prominent, exhibiting both a high enrichment factor and strong statistical significance, indicating a marked over-representation of *StbZIP* genes in transcription factor-related pathways. Moreover, enrichment of the “Signal transduction” pathway and the higher-level category “Environmental Information Processing” was evident, implicating *StbZIP* genes in signaling cascades that mediate responses to environmental stimuli. Taken together, the results support transcriptional regulation as the core functional theme of the StbZIP family, with additional involvement in biosynthetic processes, environmental responses, and signal transduction, thereby informing subsequent candidate gene validation and mechanistic pathway analyses.

### 2.6. Tissue-Specific and Low-Temperature-Responsive Expression Profiles of the StbZIP Family

**Figure 8:**
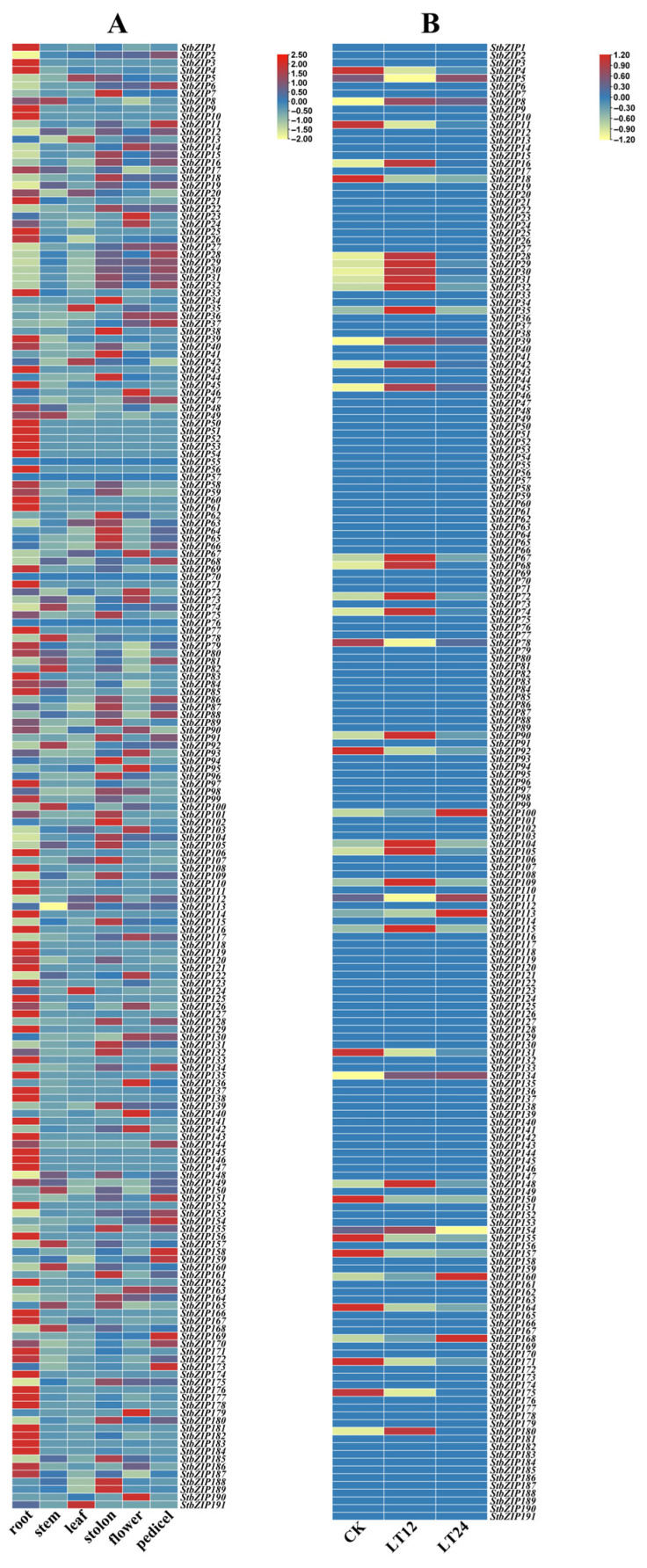
**Heatmap of expression patterns of the StbZIP family.** (**A**) The expression patterns of StbZIP genes were analyzed in six representative tissues of tetraploid potato, including the root, stem, leaf, flower, peduncle, and stolon. (**B**) The heatmap illustrates the expression patterns of *StbZIP* genes at three time points under low-temperature treatment, including 0 h, 12 h, and 24 h. The 0 h time point represents gene expression before low-temperature treatment.

Based on RNA-seq data generated from the tetraploid potato cultivar ‘Qingshu 9’, we investigated the expression profiles of StbZIP genes across different tissues and in response to low-temperature stress. (Figure 8).

The tissue-level heatmap (Figure 8A) showed that 97.91% (187/191) of *StbZIP* genes were expressed in at least one of six tissues—root, stem, leaf, flower, stolon, and flower pedicel—whereas the remaining 2.09% (4/191) exhibited no detectable expression in any tissue. Tissue specificity was quantified using the τ index (Supplementary Table S5; τ > 0.8 as the threshold), identifying 59 root-specific genes, 10 stolon-specific genes, 6 flower-specific genes, 3 flower pedicel-specific genes, and 2 leaf-specific genes.

The combined score (*D* value) (Table S4) was obtained from the five physiological indicators (Table S3) by PCA and affiliation function analysis The highest *D* value was obtained for ‘DR-2’ (0.916), indicating the strongest tolerance, whereas ‘Atlantic’ exhibited the lowest *D* value (0.142), indicating the weakest tolerance.

**Figure 9:**
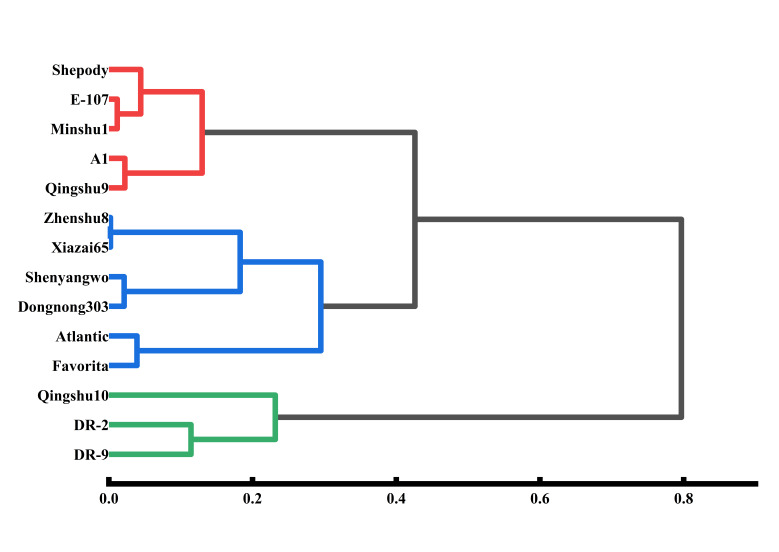
**Cluster analysis of the comprehensive cold resistance ability of potato cultivars.** Cluster analysis classified the 14 potato cultivars into three groups: strongly cold-tolerant (green), moderately cold-tolerant (red), and weakly cold-tolerant (blue) cultivars.

Cluster analysis of the comprehensive cold tolerance *D* values (Figure 9) categorized the 14 cultivars into three distinct classes (Figure 9): class I (strongly tolerant: ‘DR-2’, ‘DR-9’, and ‘Qingshu 10’); class II (moderately tolerant: ‘Qingshu 9’, ‘A1’, ‘Shepody’, ‘E-107’, and ‘Minshu1’); and class III (weakly tolerant: ‘Zhenshu8’, ‘Xiazai65’, ‘Shenyangwo’, ‘Dongnong303’, ‘Favorita’, and ‘Atlantic’).

**Figure 10:**
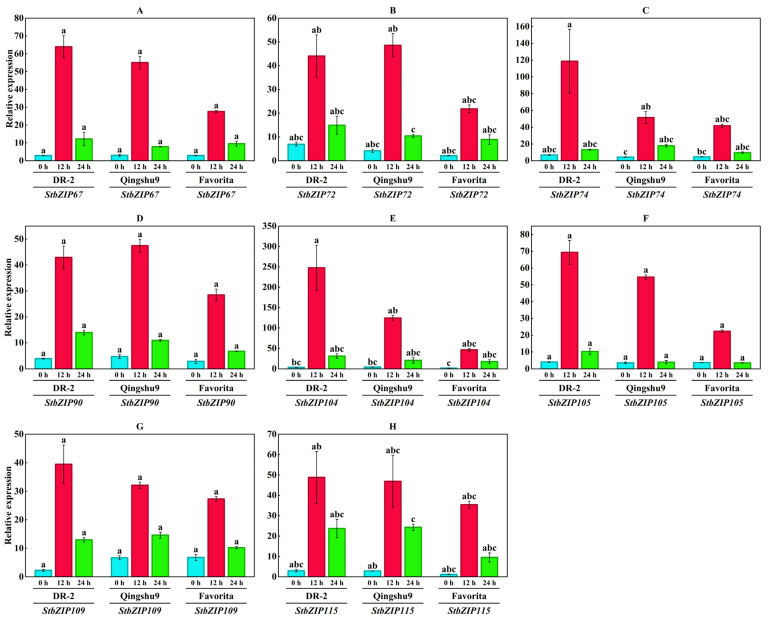
**Relative expression of representative *StbZIP* genes under cold stress.** (**A**–**H**) show the relative expression of *StbZIP67*, *StbZIP72*, *StbZIP74*, *StbZIP90*, *StbZIP104*, *StbZIP105*, *StbZIP109*, and *StbZIP115* in ‘DR-2’, ‘Qingshu 9’, and ‘Favorita’ at 0 h, 12 h, and 24 h under low-temperature treatment (4 °C), with *StActin* used as the internal reference gene. Statistical differences were assessed using a one-way Kruskal–Wallis test with Bonferroni correction, followed by Dunn’s post hoc test. Different letters indicate significant differences among groups at *p* < 0.05.

The response of the StbZIP family to low-temperature stress was assessed by analyzing expression patterns (Figure 8B). At 12 h, eight genes were significantly upregulated (log_2_FC ≥ 1 and FDR < 0.05). To validate the bioinformatic predictions, qRT-PCR was performed for the eight selected *bZIP* genes under low-temperature treatment (Figure 10). Relative expression levels were significantly elevated at 12 h and declined by 24 h, exhibiting an increase-then-decrease trajectory that was consistent with the RNA-seq-based patterns. Among the eight bZIP genes, *StbZIP104* exhibited the greatest inter-cultivar difference: at 12 h of low-temperature treatment, its relative expression in the strongly cold-tolerant cultivar ‘DR-2’ was 5.36 times that in the weakly tolerant cultivar ‘Favorita’.

### 2.7. StbZIP104 Localizes to the Nucleus

**Figure 11:**
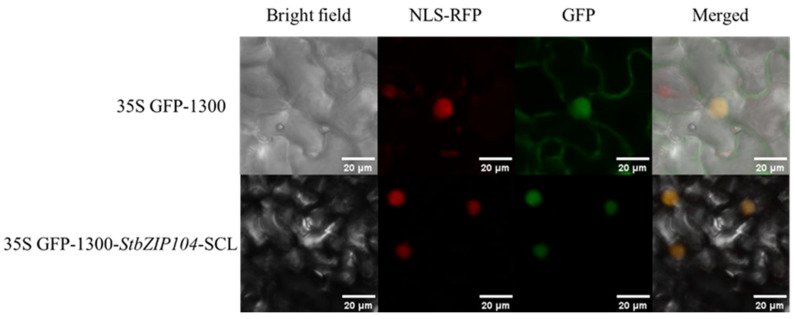
**Subcellular localization of *StbZIP104* in *Nicotiana benthamiana* leaves.** The subcellular localization of *StbZIP104* was examined by transient expression of the *StbZIP104*–GFP fusion protein in *Nicotiana benthamiana* leaves. Bright field indicates the bright-field image, NLS-RFP GFP indicates nuclear staining (red), GFP indicates the green fluorescence signal (green), and Merge represents the merged fluorescence and bright-field images (yellow). Scale bar = 20 μm.

The overlap between the GFP fluorescence signal and the nuclear localization marker indicated that *StbZIP104* was localized in the nucleus. (Figure 11)

### 2.8. Overexpression of StbZIP104 Enhances Cold Tolerance in Samsun NN Tobacco

**Figure 12:**
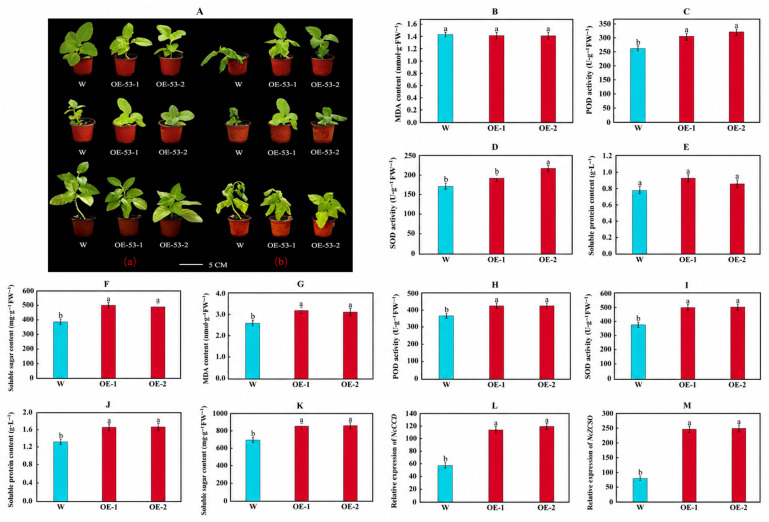
**Expression quantification of transgenic *StbZIP104*-OE lines and assessment of cold tolerance under low-temperature stress.** (**A**) Phenotypes of wild-type and overexpression lines before and after low-temperature stress. (**a**) Plants before low-temperature stress; (**b**) plants after low-temperature stress. Scale bars = 5 cm. (**B**–**F**) Physiological indices of wild-type (W) and transgenic Samsun NN tobacco lines before cold stress. (**G**–**K**) Physiological indices of wild-type (W) and transgenic Samsun NN tobacco lines after cold stress. (**L**,**M**) Relative expression of *NtCSD* and *NtFSD* in wild-type (W) and *StbZIP104*-overexpressing Samsun NN tobacco plants after 12 h at 4 °C, with *NtActin* used as the internal reference gene. Statistical differences were assessed using a one-way Kruskal–Wallis test with Bonferroni correction, followed by Dunn’s post hoc test. Different letters indicate significant differences among groups at *p* < 0.05.

Considering the phylogenetic relationship between potato and tobacco within the Solanaceae family, tobacco was employed as a heterologous expression system to preliminarily assess the function of *StbZIP104*. To assess the function of *StbZIP104* under low-temperature stress, wild-type (WT) plants and two independent *StbZIP104* overexpression lines (*StbZIP104*-OE-1 and *StbZIP104*-OE-2) were placed in a 4 °C chamber for 24 h. After treatment, WT plants exhibited more pronounced wilting than the overexpression lines (Figure 12A). Physiological indices were then quantified under cold stress. Compared with WT, the overexpression lines showed significantly higher peroxidase (POD) and superoxide dismutase (SOD) activities and increased contents of soluble protein and soluble sugars (Figure 12B–K), indicating enhanced osmotic adjustment and reactive oxygen species (ROS) scavenging capacity. Conversely, malondialdehyde (MDA) content was significantly lower in the overexpression lines, consistent with reduced membrane lipid peroxidation. Taken together, these results demonstrate that *StbZIP104* overexpression confers increased cold tolerance in Samsun NN tobacco.

**Figure 13:**
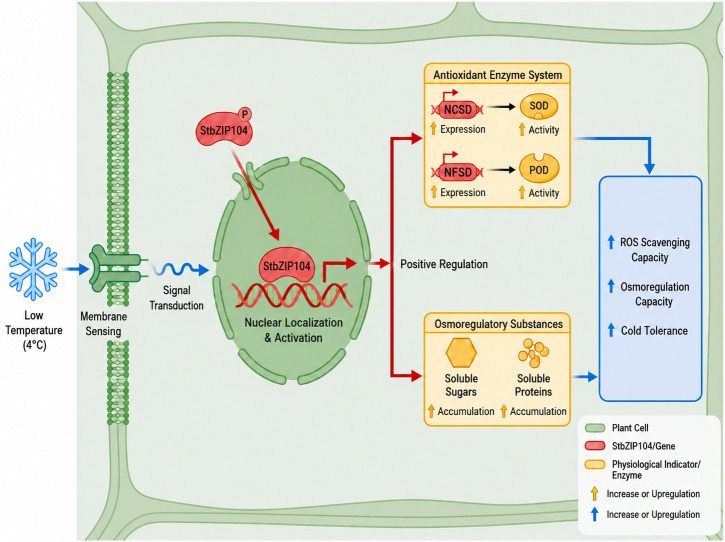
**Schematic illustration of the proposed role of *StbZIP104* in regulating plant cold tolerance under low-temperature stress.** Low temperature (4 °C) is perceived by the cell membrane and triggers intracellular signal transduction, leading to the activation and nuclear localization of StbZIP104. Activated *StbZIP104* positively regulates the antioxidant enzyme system and osmotic adjustment, as reflected by the increased expression of *NtCSD* and *NtFSD*, enhanced SOD and POD activities, and increased accumulation of soluble sugars and soluble proteins. These changes contribute to improved ROS-scavenging capacity, enhanced osmoregulatory capacity, and ultimately increased cold tolerance. Red arrows indicate positive regulation or activation, blue arrows indicate the progression of the response process and enhanced physiological effects, and black arrows indicate the direct relationship between biological events.

The relative expression of *NtCSD* and *NtFSD* was quantified in wild-type (W) and *StbZIP104*-overexpressing Samsun NN tobacco plants after 12 h of cold treatment (4 °C) to preliminarily infer downstream targets of *StbZIP104* (Figure 12L,M). Both *NtCSD* and *NtFSD* were significantly upregulated in *StbZIP104*-overexpressing plants compared with W under cold stress, suggesting that *StbZIP104* may enhance cold tolerance by modulating the ROS regulatory pathway (Figure 13).

## 3. Discussion

The cold tolerance of plants is a complex regulatory process jointly controlled by multiple genes. Therefore, reliance on a single parameter, such as antioxidant enzyme activity or osmotic adjustment substance content, is insufficient for a comprehensive evaluation of plant cold tolerance. Moreover, individual physiological indicators may contribute differently to the low-temperature response of potato, indicating that cold tolerance in potato is a complex quantitative trait that cannot be accurately or intuitively assessed using a single index alone. To overcome the limitations of single-indicator evaluation, it is necessary to integrate multivariate statistical approaches for further analysis. Accordingly, in this study, principal component analysis and the membership function method, two widely used approaches for the comprehensive evaluation of plant stress resistance, were employed to improve the objectivity and accuracy of cold tolerance assessment. It should be noted that cold tolerance grading was performed under controlled low-temperature conditions using tissue culture-derived potato plantlets; thus, the results reflect seedling-stage responses and lack validation against field performance under natural cold stress.

The basic region/leucine zipper (bZIP) transcription factor family is widely involved in regulating plant growth, development, and responses to environmental stimuli. In Arabidopsis thaliana, *bZIP* genes are implicated in diverse processes, including abiotic stress responses, light signaling and morphogenesis, and seed germination [12,13,14]. However, research on this family in Solanaceae plants, particularly in potato, remains limited. Most potato gene family analyses have focused on diploid reference genomes, whereas widely cultivated potatoes are typically tetraploid and exhibit high heterozygosity, making genetic and genomic analyses more challenging. Consequently, genome-wide characterization of the bZIP family in tetraploid potato has lagged behind. The recent release of high-quality tetraploid potato genomes, including the C88.v1 assembly and the tetraploid cultivar ‘Qingshu 9’ [15,16], has enabled comprehensive bioinformatic identification and analysis of *bZIP* genes at the whole-genome level.

In the cultivated tetraploid potato ‘Qingshu 9’, 191 *bZIP* genes were identified (Table S1), far exceeding the numbers reported for tomato (69) [17] and diploid potato (49) [18]. This disparity is likely attributable to polyploidy-associated effects in the tetraploid potato and expansion of the bZIP family [19,20]. The StbZIP family was classified into 11 subfamilies (A–I, S, and U), largely paralleling the scheme established for Arabidopsis thaliana, indicating interspecific conservation; most subfamilies contain orthologous members across species, reflecting evolutionary continuity [21]. Notably, subfamily U was observed exclusively in the tetraploid potato and had no counterpart in Arabidopsis, suggesting a lineage-specific expansion in potato that may confer species-specific functions. At the chromosomal level, multiple *StbZIP* genes were closely clustered on chromosomes such as Chr01A4, Chr03A4, Chr04A3, and Chr10A, indicating the possible existence of tandem replication expansion events.

Intraspecific collinearity analysis indicated that whole-genome duplication (WGD) and segmental duplication were the predominant drivers of StbZIP family expansion, consistent with evolutionary patterns reported for most plant gene families [22]. Analysis of Ka/Ks across all syntenic pairs showed that the mean Ka/Ks value of tandem duplicates was slightly higher than that of WGD/segmental duplicates, suggesting that both classes have been shaped mainly by purifying selection, but that tandem duplicates experience relatively relaxed selective constraints [23]. From a cross-species synteny perspective, tetraploid potato *bZIP* genes retained the highest number of syntenic homologs in tomato, whereas the number of syntenic homologs was slightly reduced in Arabidopsis thaliana. This number declined markedly in the monocots rice and maize, a pattern consistent with synteny analyses of multiple gene families in potato [24,25].

Analyses of gene structure, conserved domains, and conserved motifs further corroborated the phylogenetic classification of the StbZIP family [26,27]. Members within the same subfamily typically exhibited similar exon–intron organizations and domain/motif architectures. This conservation is indicative of descent from a common ancestor and suggests potential functional overlap or cooperativity among subfamily members [28]. By analyzing the conserved domain, the potential functions of the *StbZIP* genes can be preliminarily inferred. For instance, the DOG1 superfamily is related to the regulation of seed dormancy [29]; thus, the D subfamily may be involved in the regulation of seed dormancy.

Promoter analysis predicted cis-acting elements for the *StbZIP* genes, with a marked enrichment of ABA-responsive motifs indicating broad involvement of the potato bZIP family in ABA-mediated transcriptional regulation. Furthermore, 73.82% of *StbZIP* gene promoters harbored hormone-, stress-, and light-responsive elements, indicating that the majority of *StbZIP* genes are subject to complex multi-signal regulation and likely function at the interface of signal transduction and environmental adaptation [30]. Consistently, GO and KEGG enrichment analyses corroborated these patterns.

The analysis of the expression patterns of the *StbZIP* genes in different tissues and under low-temperature stress further highlights the diversity and specificity of the *StbZIP* genes’ functions. Analysis of tissue expression patterns revealed that 97.91% (187/191) of *StbZIP* genes exhibited detectable expression in at least one of six tissues: root, stem, leaf, flower, stolon, and pedicel. Among these, 80 genes showed tissue-specific expression. The observed spatial differentiation in expression is indicative of specialized, tissue-specific functions of distinct *StbZIP* genes in potato growth and development [31]. Expression analysis under low-temperature stress revealed that most *StbZIP* genes were minimally affected by short-term treatment, whereas eight members were significantly upregulated at 12 h. These low-temperature-induced *StbZIP* genes exhibited a transient induction, increasing initially and subsequently declining. This dynamic expression trajectory suggests that these *StbZIP* genes act as upstream responders involved in cold-signal perception and transduction in potato, thereby initiating downstream defense programs during the early phase of stress [32]. Among the cold-induced *StbZIP* genes, *StbZIP104* exhibited the most pronounced differential expression: its transcript level in the strongly cold-tolerant cultivar ‘DR-2’ was 5.36-fold higher than in the weakly tolerant cultivar ‘Favorita’, suggesting an important role for *StbZIP104* in potato cold tolerance.

Following exposure to low-temperature stress, plants mount a suite of physiological and biochemical responses. Numerous studies have shown that cold tolerance is closely associated with the extent of membrane lipid peroxidation, the activities of antioxidant enzymes, and the accumulation of osmolytes. Exposure to low-temperature stress can trigger cellular oxidative stress, resulting in the excessive accumulation of reactive oxygen species (ROS) and malondialdehyde (MDA), which further promotes membrane lipid peroxidation and oxidative injury [33,34]. In this study, *StbZIP104* overexpression lines exhibited significantly lower MDA levels than the wild type under low-temperature stress, consistent with enhanced resistance of cellular membranes to oxidative injury. To mitigate ROS stress, plants deploy both enzymatic and non-enzymatic antioxidant defenses. The non-enzymatic arm includes antioxidants such as reduced glutathione and ascorbate [35,36]. The enzymatic arm encompasses superoxide dismutase (SOD) and peroxidases (PODs), among others. SOD catalyzes the dismutation of superoxide (O_2_•^−^) to H_2_O_2_ and O_2_, thereby limiting superoxide-mediated damage [37], whereas POD decomposes H_2_O_2_, safeguarding cellular membranes from peroxidative injury [38]. Furthermore, the activities of SOD and POD under low-temperature stress were markedly elevated in *StbZIP104* overexpression lines relative to the wild type, suggesting an improved capacity for ROS scavenging in the transgenic plants. Under low-temperature stress, plants accumulate soluble sugars and soluble proteins, which serve as compatible solutes (osmolytes) to stabilize cellular osmotic potential, mitigate dehydration, and potentially act as signaling molecules that regulate cold-responsive pathways [39]. Here, the contents of soluble proteins and soluble sugars were significantly elevated in *StbZIP104* overexpression lines compared to the wild type under low-temperature conditions, indicating enhanced osmotic adjustment capacity in the transgenic plants. Combined with the antioxidant data, these findings suggest that *StbZIP104* enhances cold tolerance by coordinately improving ROS detoxification and strengthening osmotic regulation.

The ROS regulatory pathway is widely regarded as a core module in plant stress-response networks. Previous studies have shown that *GhbZIP53* enhances stress tolerance in cotton, at least in part, by upregulating *GhSOD* [40] and that *OsbZIP53* contributes to rice disease resistance by modulating SOD activity [41]. In the present study, qRT-PCR analysis showed that the transcript levels of *NtCSD* and *NtFSD* were significantly higher in *StbZIP10*4-overexpressing Samsun NN tobacco plants than in the wild type after 12 h of cold treatment at 4 °C. These results suggest that *StbZIP104* may be involved in ROS-scavenging-related responses under cold stress, which may partly account for the enhanced cold tolerance observed in the overexpression lines. However, the current evidence is mainly based on the expression patterns of ROS-scavenging-related genes in a heterologous tobacco system, and direct evidence demonstrating that *StbZIP104* regulates ROS homeostasis remains limited. Therefore, future studies involving loss-of-function analysis of *StbZIP104* and identification of its downstream target genes will be necessary to further elucidate the regulatory network through which *StbZIP104* participates in plant responses to cold stress.

## 4. Materials and Methods

### 4.1. Identification of the StbZIP Family and Analysis of Physicochemical Properties

Genome and protein sequence data for the tetraploid potato cultivar ‘Qingshu 9’ were downloaded from the National Genomics Data Center (https://ngdc.cncb.ac.cn/, accessed on 25 June 2025) [16]. Hidden Markov model (HMM) profiles for the bZIP_1 and bZIP_2 domains (Pfam accessions PF00170 and PF07716) were retrieved from the InterPro database (https://www.ebi.ac.uk/interpro/, accessed on 14 July 2025) [42]. To screen putative members of the StbZIP family, the predicted proteome of ‘Qingshu 9’ was searched against these domain profiles using HMMER v3.0 with the hmmsearch program, applying an E-value cutoff of <1.0 [43]. The resulting candidate proteins were further examined using the NCBI Conserved Domain Database (CDD, https://www.ncbi.nlm.nih.gov/Structure/cdd/cdd.shtml, accessed on 14 July 2025) [44] to verify the presence of the canonical bZIP domain. The chromosomal locations of the identified StbZIP genes were mapped and visualized using TBtools-II [45]. Additionally, protein length, molecular weight, and pI were determined with TBtools-II. The forecasted subcellular localization of StbZIP proteins was evaluated employing DeepLoc 2.0 [46] (https://services.healthtech.dtu.dk/services/DeepLoc-2.0/, accessed on 14 July 2025).

### 4.2. Phylogenetic Tree Construction and Classification of the StbZIP Family

Protein sequences of Arabidopsis bZIP members were retrieved from the TAIR database [47] (https://www.arabidopsis.org/, accessed on 14 July 2025). Multiple sequence alignment between AtbZIP and StbZIP proteins was performed in MEGA v12.0 [48], and a maximum-likelihood phylogenetic tree was subsequently generated. Based on the subfamily classification framework proposed by Jakoby [6], the identified StbZIP proteins were assigned to corresponding subfamilies.

### 4.3. Gene Structure, Conserved Domain, and Conserved Motif Analyses of the StbZIP Family

To characterize gene structure, the coding sequences of StbZIP genes from ‘Qingshu 9’ were aligned with their corresponding genomic sequences using TBtools-II, allowing exon–intron arrangements to be examined. Conserved motifs in the StbZIP proteins were identified through the MEME Suite online program [49] (http://meme-suite.org/, accessed on 14 July 2025). The motif distribution patterns were subsequently displayed with Evolview v2 [50] (https://evolgenius.info/evolview-v2/, accessed on 20 August 2025).

### 4.4. Promoter Cis-Acting Element Analysis of the StbZIP Family

The 2000 bp sequences upstream of each StbZIP gene were retrieved using TBtools-II and regarded as putative promoter regions. The categories and numbers of cis-acting regulatory elements within these promoter sequences were predicted with PlantCARE [51] (https://bioinformatics.psb.ugent.be/webtools/plantcare/html/, accessed on 14 July 2025). The resulting distribution patterns of promoter cis-elements across the StbZIP family were further visualized using Evolview v2 (https://evolgenius.info/evolview-v2/, accessed on 20 August 2025).

### 4.5. Collinearity Analysis of the StbZIP Family

Intraspecific and interspecific collinearity were assessed using MCScanX [52] as implemented in TBtools-II, and synonymous and nonsynonymous substitution rates, together with Ka/Ks values, were calculated using TBtools-II [24].

### 4.6. Functional Prediction of the StbZIP Family

Gene Ontology (GO) and KEGG pathway annotations for the StbZIP family were obtained using EGGNOG-MAPPER v2 [53] (http://eggnog-mapper.embl.de/, accessed on 14 July 2025) and visualized in TBtools-II.

### 4.7. Analysis of the Expression Patterns of the StbZIP Family and Verification by qRT-PCR in Different Cold-Resistant Potato Varieties

#### 4.7.1. Expression Pattern Analysis of the StbZIP Family

RNA-seq datasets for the root, stem, leaf, flower, stolon, and flower pedicel were obtained from the National Genomics Data Center (https://ngdc.cncb.ac.cn/, accessed on 25 June 2025) [16]. Leaf transcriptomes under low-temperature stress (4 °C, 24 h) were retrieved from the same resource.

The tissue specificity of *StbZIP* genes was assessed using the tau (τ) index. The τ index ranges from 0 to 1; values near 0 suggest widespread expression across all examined tissues, while values approaching 1 indicate highly tissue-specific expression. In this study, genes with τ > 0.8 were classified as tissue specific, whereas those with τ < 0.2 were considered ubiquitously expressed [54].

#### 4.7.2. Screening of Potato Cultivars with Contrasting Cold Tolerance Using Physiological Indices

Plant cold tolerance is typically evaluated through field natural frost assays [55], physiological and biochemical measurements [56], and electrolyte leakage tests [57]. Notably, the physiological–biochemical index method incorporates multiple parameters and, when paired with principal component analysis (PCA) and membership function scoring, offers a thorough assessment that effectively differentiates cultivar variations in cold tolerance with considerable accuracy.

Tissue-cultured plantlets from fourteen cultivars—‘Shepody’, ‘E-107’, ‘Minshu1’, ‘Qingshu 10’, ‘Zhenshu8’, ‘A-1’, ‘DR-2’, ‘DR-9’, ‘Atlantic’, ‘Xiazai65’, ‘Qingshu 9’, ‘Favorita’, ‘Shenyangwo’, and ‘Dongnong303’—were selected for cold tolerance screening (Table 1). Experimental materials were provided by the Institute of Biotechnology, Qinghai Academy of Agriculture and Forestry Sciences.

Tissue-cultured plantlets of the 14 cultivars were grown on Murashige and Skoog (MS) medium [58] for 20 days, after which plantlets at a comparable developmental stage were selected for subsequent experiments. The treatment group was subjected to low-temperature stress at 4 °C under 10,000 lx illumination with a 16 h photoperiod at a relative humidity of 60%, with five independent biological replicates. The control group was maintained in a growth chamber at 21 °C under identical conditions. Plantlets were sampled at 0, 1, 3, 5, and 7 d after treatment for subsequent analyses. Contents of MDA [59], soluble sugars [60], and soluble proteins [61], as well as activities of POD [62] and SOD [63], were measured utilizing commercial assay kits (Nanjing Jiancheng Bioengineering Institute, Nanjing, China) in accordance with the provided guidelines.

Data were compiled in Microsoft Excel 2022. PCA was performed in IBM SPSS Statistics 27, and clustering analyses were conducted in Origin 2025. Relevant indices were calculated following Han [56].

Membership function value for each indicator:

(1) U(X_j_) = (X_j_ − X_min_)/(X_max_ − X_min_) j = 1, 2, 3, …, n

Weight of each composite indicator:

(2) $$W_{j}=p_{j}/\sum_{j=1}^{n}p_{j}\;j=1,2,3,\ldots ,n$$

Comprehensive cold tolerance value D for each potato cultivar:

(3) $$D=\sum_{j=1}^{n}\left[U(X_{j})\times W_{j}\right]\;j=1,2,3,\ldots ,n$$

In Equations (1)–(3), $X_{j}$ indicates the value of the jth evaluation index, whereas $X_{min}$ and $X_{max}$ correspond to the lowest and highest values of this index, respectively. $W_{j}$ represents the weighting coefficient of the jth comprehensive indicator, reflecting its relative contribution among all comprehensive indicators. $P_{j}$ denotes the contribution rate of the jth comprehensive indicator for each potato genotype. The *D* value represents the comprehensive evaluation value of cold tolerance for each potato material under low-temperature stress, calculated based on the comprehensive indicators. When 0.7 ≤ *D* ≤ 1, the material is classified as a strongly cold-tolerant cultivar; when 0.4 ≤ *D* ≤ 0.69, it is classified as a moderately cold-tolerant cultivar; and when 0 ≤ *D* ≤ 0.39, it is classified as a weakly cold-tolerant cultivar.

After the SOD activity, POD activity, soluble sugar content, soluble protein content, and MDA content of each material were standardized using Equation (1) in the data-processing procedure, principal component analysis was performed on the standardized data to generate five composite indicators. Composite indicators with a cumulative contribution rate greater than 85% were retained for subsequent analysis. The weight (W) of each selected composite indicator was then calculated according to Equation (2). Finally, the membership function value of each composite indicator and the comprehensive cold tolerance evaluation value (*D*) of each material were calculated using Equations (1) and (3), respectively.

#### 4.7.3. qRT-PCR Validation of StbZIP Genes in Potato Cultivars with Contrasting Cold Tolerance

qRT-PCR was performed using three cultivars representing contrasting tolerance: strongly tolerant ‘DR-2’, moderately tolerant ‘Qingshu 9’, and weakly tolerant ‘Favorita’. Low-temperature treatment followed Section 4.7.2 (4 °C for 24 h). Leaf samples were collected at 0, 12, and 24 h and promptly subjected to RNA extraction. Total RNA was isolated from leaf tissues collected at the designated sampling points using a plant RNA extraction kit (Takara Biotech, Beijing, China). The purified RNA was then converted into cDNA with a reverse transcription kit following the protocol provided by the manufacturer (Takara Biotech, Beijing, China) [64]. QRT-PCR was conducted with TB Green Premix Ex Taq II (Takara Biotech, Beijing, China) in accordance with the provided guidelines. [65]. Thermal cycling on a Roche LightCycler^®^ 96 (Beijing, China) consisted of 95 °C for 30 s, followed by 40 cycles of 95 °C for 10 s and 57 °C for 32 s, and a final dissociation/melting-curve step of 95 °C for 60 s, 57 °C for 60 s, and 55 °C for 10 s. *StActin* [66] served as the internal reference gene. Each sample was analyzed with three biological replicates and three technical replicates to determine relative expression levels utilizing the 2^−ΔΔCt^ method [67]. The qRT-PCR primers were designed through NCBI Primer-BLAST, and detailed primer sequences can be found in Table S8.

### 4.8. Subcellular Localization of StbZIP104

The *StbZIP104* coding region without the termination codon was amplified using specific primers, and the amplification product was subsequently verified by PCR. The purified PCR fragment was subsequently ligated into the 35S-GFP-1300-RUAN′ vector using the Acc65I and SalI restriction sites, resulting in the recombinant plasmid 35S-GFP-1300-RUAN′-*StbZIP104*. After the sequence was verified, the recombinant plasmid was introduced into Agrobacterium tumefaciens strain GV3101 (pSoup-p19). To conduct transient expression assays, 5 mL of A. tumefaciens suspension harboring the desired construct was inoculated into 50 mL of LB broth supplemented with kanamycin and rifampicin. The culture was then cultivated at 28 °C with agitation at 200 rpm until reaching an OD600 of 0.8–1.0. The cells were harvested and resuspended in MES buffer, and the bacterial suspension was standardized to an OD600 of 0.6–0.8. Acetosyringone was added at a 1:1000 dilution to reach a final concentration of 100 μM, followed by thorough mixing. The suspensions containing the marker construct and the target fusion construct were then combined at a 1:3 ratio and utilized for leaf infiltration. *Nicotiana benthamiana* plants aged five to six weeks, exhibiting uniform growth, were chosen and subjected to 2–3 h of incandescent light exposure to induce stomatal opening. The infiltration mixture was carefully applied to the underside of *Nicotiana benthamiana* leaves using a 5 mL needleless syringe, ensuring avoidance of the primary veins. Subsequently, the infiltrated plants were kept in darkness for 24 h initially and then transferred to a light incubator for an additional 48–72 h [68]. Fluorescence signals were observed with a laser scanning confocal microscope. Epidermal cells were excited at 514 nm, and the emitted fluorescence was recorded within the wavelength range of 524–574 nm.

### 4.9. Functional Validation of StbZIP104

The coding sequence of *StbZIP104* was acquired through PCR amplification using gene-specific primers and then inserted into the PRI101 expression vector via the Acc65I and SalI restriction sites, resulting in the PRI101-*StbZIP104* construct. Following confirmation, this construct was introduced into A. tumefaciens strain GV3101(pSoup-p19). The Agrobacterium strain was cultured in LB broth supplemented with rifampicin and kanamycin until reaching an OD600 of about 0.8. Samsun NN tobacco plants underwent genetic transformation through the leaf-disc technique facilitated by A. tumefaciens [69]. After co-cultivation, the infected leaf discs were moved onto selective regeneration medium to promote shoot formation. Newly regenerated shoots were then transferred to rooting medium, and candidate transgenic plants were recovered under antibiotic selection. PCR analysis was used to confirm the presence of StbZIP104 in the regenerated plants, while qRT-PCR was performed to evaluate its transcript level in positive lines. After propagation to the T2 generation, the confirmed transgenic tobacco lines were subjected to low-temperature stress treatments and subsequent physiological analyses.

Wild-type and transgenic tobacco plantlets were cultured in vitro for 20 d, transplanted to 6 cm pots, and acclimated for 7 d before treatment. Low-temperature stress was imposed at 4 °C under 10,000 lx with a 12 h light/12 h dark photoperiod at a relative humidity of 60%, and plants were photographed after 1 d of treatment. Control plants were maintained at 21 °C under identical conditions (10,000 lx; 12 h light/12 h dark; 60% relative humidity). Samples were collected at 0, 12, and 24 h for qRT-PCR analysis and physiological parameter measurements.

The qRT-PCR procedure was the same as described in Section 4.7.3. *NtActin* was used as the internal reference gene, and all primer sequences are listed in Supplementary Table S8.

Contents of MDA [59], soluble sugars [60], and soluble proteins [61], as well as activities of POD [62] and SOD [63], were measured utilizing commercial assay kits (Nanjing Jiancheng Bioengineering Institute, Nanjing, China) in accordance with the provided guidelines.

## Figures and Tables

**Table 1:** Source and maturity characteristics of potato germplasm resources.

Germplasm Resources	Maturity
Shepody	Mid-maturing
E-107	-
Minshu1	Mid-maturing
Qingshu10	Late maturing
Zhenshu8	Early maturing
A1	-
DR-2	-
DR-9	-
Atlantic	Mid-maturing
Xiazai65	Mid-maturing
Qingshu9	Late maturing
Favorita	Early maturing
Shenyangwo	Mid-maturing
Dongnong303	Early maturing

## Data Availability

The genome datasets and transcriptome datasets used in this study are available in the National Genomics Data Center (http://bigd.big.ac.cn/, accessed on 25 June 2025). The autotetraploid potato genome sequence and resequencing data have been deposited in the Genome Sequence Archive at the National Genomics Data Center and are accessible at http://bigd.big.ac.cn/ (accessed on 25 June 2025) under BioProject numbers PRJCA006096 and PRJCA006099, respectively. The gene expression matrices have also been deposited in the Genome Sequence Archive and are accessible at http://bigd.big.ac.cn/ (accessed on 25 June 2025) under the BioProject number PRJCA006877. All data are available upon request to the corresponding author.

## Supplementary Materials

The following supporting information can be downloaded at: https://www.mdpi.com/article/10.3390/plants15101513/s1: Table S1. StbZIP family identification and physicochemical properties analysis. Table S2. Duplication types and Ka/Ks ratios of *StbZIP* genes. Table S3. Results of physiological indices of different potato cultivation resources under cold stress. Table S4. Comprehensive index values, weights, u(X), and D values of different potato cultivation. Table S5. Expression patterns of *StbZIP* genes across tissues in the potato cultivar ‘Qingshu 9’. Table S6. Cold-responsive expression of StbZIP family genes. Table S7. Cis-acting elements of *StbZIP* genes in potato. Table S8. All primer sequence information used in this article. Table S9. Plant species and genotypes used in this study and their applications.
